# Hailey-Hailey Disease is Associated with Diabetes: A Population-based Cohort Study, Clinical Cohort Study, and Pedigree Analysis

**DOI:** 10.2340/actadv.v103.10436

**Published:** 2023-11-28

**Authors:** Philip CURMAN, William JEBRIL, Carmella EVANS-MOLINA, Etty BACHAR-WIKSTROM, Henrik LARSSON, Martin CEDERLÖF, Jakob D. WIKSTROM

**Affiliations:** 1 Dermatology and Venereology Division, Department of Medicine (Solna), Karolinska Institutet; 2 Dermato-Venereology Clinic, Karolinska University Hospital; 3 Department of Medical Epidemiology and Biostatistics, Karolinska Institutet, Stockholm, Sweden; 4 Departments of Anatomy, Cell Biology, and Physiology; 5 Biochemistry and Molecular Biology; 6 Biochemistry and Medicine; 7 Biochemistry and Pediatrics; 8 The Center for Diabetes and Metabolic Diseases; 9 Herman B Wells Center for Pediatric Research, Indiana University School of Medicine; 10 Roudebush VA Medical Center, Indianapolis, IN, USA; 11 School of Medical Sciences, Faculty of Medicine and Health, Örebro University, Örebro, Sweden

**Keywords:** Hailey-Hailey disease, diabetes, SPCA1, human leukocyte antigen, pedigree, cohort study

## Abstract

Hailey-Hailey disease is a rare hereditary skin disease caused by mutations in the *ATP2C1* gene encoding the secretory pathway Ca^2+^/Mn^2+^-ATPase 1 (SPCA1) protein. Extracutaneous manifestations of Hailey-Hailey disease are plausible but still largely unknown. The aim of this study was to explore the association between Hailey-Hailey disease and diabetes. A population-based cohort study of 347 individuals with Hailey-Hailey disease was performed to assess the risks of type 1 diabetes and type 2 diabetes, using Swedish nationwide registries. Pedigrees from 2 Swedish families with Hailey-Hailey disease were also investigated: 1 with concurrent type 1 diabetes and HLA-DQ3, the other with type 2 diabetes. Lastly, a clinical cohort with 23 individuals with Hailey-Hailey disease and matched healthy controls was evaluated regarding diabetes. In the register data males with Hailey-Hailey disease had a 70% elevated risk of type 2 diabetes, whereas no excess risk among women could be confirmed. In both pedigrees an unusually high inheritance for diabetes was observed. In the clinical cohort, individuals with Hailey-Hailey disease displayed a metabolic phenotype indicative of type 2 diabetes. Hailey-Hailey disease seems to act as a synergistic risk factor for diabetes. This study indicates, for the first time, an association between Hailey-Hailey disease and diabetes and represents human evidence that SPCA1 and the Golgi apparatus may be implicated in diabetes pathophysiology.

SIGNIFICANCE

This study showed, for the first time, a manifestation of the severe skin disorder Hailey-Hailey disease outside the skin. By studying 2 Swedish nationwide registers, 2 family pedigrees with concurrent diabetes type 1 and 2, and a clinical cohort with matched healthy controls, the results show that there is an association between Hailey-Hailey disease and diabetes. This information potentially has great impact for the care and management of patients with Hailey-Hailey disease and provides interesting insight into the multi-faceted pathophysiology of diabetes.

Hailey-Hailey disease (HHD), also known as familial benign chronic pemphigus, is a rare autosomal dominant genodermatosis characterized by persistent blisters and erosions, typically occurring in skin folds. Symptoms are chronically relapsing and often exacerbated by secondary bacterial infection, with disease onset in the third or fourth decade (1). HHD is caused by heterozygous mutations in the *ATP2C1* gene, which encodes the human secretory pathway Ca^2+^/Mn^2+^-ATPase 1 (SPCA1) protein (2). This mutation gives rise to cellular Ca^2+^ dyshomeostasis, as SPCA1 transports Ca^2+^ into the Golgi apparatus, an essential organelle for intracellular Ca^2+^ storage. The disease phenotype of HHD is caused by *ATP2C1* haploinsufficiency and biallelic mutations are lethal (3). Mutations in the *ATP2C1* gene have been shown to produce dysfunctional proteins, that may be degraded and impair normal cellular functions, resulting in abnormal keratinocyte adhesion in the suprabasal layer of the epidermis, a feature termed acantholysis. To date, more than 200 different *ATP2C1* mutations that result in HHD have been identified, including frameshift, missense, splice site, and nonsense variants (4). SPCA1 is ubiquitously expressed in all cells, rendering it plausible to suspect extracutaneous manifestations in HHD (2, 5).

The 2 most common types of diabetes mellitus (6) are type 1 diabetes (T1D) and type 2 diabetes (T2D). T1D is caused by autoimmune destruction of the pancreatic β-cells, leading to hyperglycaemia due to severe reductions in insulin secretion. While the peak incidence of T1D occurs during childhood, approximately half of all new diagnoses of T1D occur in adults (7). The underlying trigger for autoimmunity in T1D is not fully established; however, genetics probably play a major part. Variations in the HLA region are the strongest genetic determinant of T1D risk with the highest risk loci being those that encode HLA-DQ3 and HLA-DR4 (8). In addition to HLA risk, more than 40 different non-human leukocyte antigen (HLA) risk regions for T1D have been described (9). Since the incidence of T1D varies between countries and ethnicities, environmental factors might also contribute to disease development (10). T2D is the most common form of diabetes and carries a stronger genetic predisposition than T1D (11). The primary underlying cause of T2D is obesity-associated insulin resistance, which later progresses to β-cell failure and an insulin-deficient state. T2D has historically been viewed as a disease of adults; however, in parallel with increasing rates of obesity across the lifespan, T2D is increasingly diagnosed in children (12). Despite much research, the pathophysiology of T1D and T2D is incompletely understood.

Monogenic human diseases, similar to transgenic animal models, enable the study of specific candidate disease genes relevant not only for the rare disease itself, but also for more common conditions (13). Based on our clinical observations as well as the preclinical literature highlighting a role for *SPCA1* in β-cell function and diabetes pathophysiology (14), we hypothesized that there is an association between HHD and diabetes and that HHD may act as a synergistic risk factor for the development of diabetes. The current study tests this hypothesis in a population-based cohort, a clinical HHD cohort, and pedigree analyses of families with HHD and concomitant T1D and T2D.

## MATERIALS AND METHODS

### Population-based cohort study

A national, register-based, cohort study was performed to assess the risks of T1D, T2D, and “other specified diabetes mellitus” in individuals with HHD. Linkage between registers was made possible by the personal identification number, assigned at birth or at immigration to Sweden. The Total Population Register was used to extract information about the birth year and sex of the participants, the National Patient Register (NPR) (15), which contains discharge diagnoses assigned by the treating medical doctor in inpatient care, and outpatient hospital visits, according to the International Classification of Diseases, Tenth Revision (ICD-10) (16), which was launched in 1997, and the Cause of Death Register (17). The end of data coverage was 31 December 2013, meaning that the study spanned 16 years. A validation study has shown that 85–95% of diagnoses of chronic disorders, such as HHD, in the NPR are valid (15). HHD was defined as an ICD-10 code of Q82.8D; T1D was defined as E10, T2D according to E11, and other specified diabetes as E13. The first of the participants’ respective diagnoses were used in the analyses. Comparison subjects were selected randomly from the general population on a 1:100 ratio and matched for age and sex.

### Family/pedigree studies

The study included pedigrees from two Swedish families with HHD; 1 with concurrent T1D and HLA-DQ3 mutations, the other with concurrent T2D. To evaluate the family history of the T1D family, the study gathered information through patient history at the Department of Dermato-Venereology at Karolinska University Hospital, Stockholm, Sweden. For the family with HHD and concurrent T2D, all information was gathered from two index patients and through medical and health records.

### Clinical cohort study

Twenty-three individuals with HHD were matched with 23 healthy controls in a 1:1 ratio regarding age (± 5-year intervals), sex, and body mass index (BMI) (< 18.5, 18.5–24.99, 25–29.99 and > 30 kg/m^2^) (**Table I**). Inclusion criteria were a diagnosis of HHD by a dermatologist based on typical clinical appearance, family history, and histopathology. Exclusion criteria were age < 18 years, current pregnancy, active substance abuse, or acute illness in the past four weeks. Peripheral venous blood for diabetes metabolic parameters were collected. Specifically, we measured the variables Homeostasis Model Assessment (HOMA)2-%S and HOMA2-%B, markers for insulin resistance and β-cell dysfunction, respectively, since HOMA2-%B was reported to be increased in Darier disease (DD) (18). DD is a skin condition with similar pathophysiology to HHD, though with a dysfunction in the endoplasmic reticulum rather than the Golgi apparatus. Insulin was analysed using Cobas Elecsys Insulin immunoassay (Roche, Basel, Switzerland) and proinsulin using Mercodia Proinsulin ELISA (Mercodia, Uppsala, Sweden).

**Table I:** Baseline characteristics and diabetes-related metabolic parameters of participant individuals with Hailey-Hailey disease (HHD) and healthy matched controls in the clinical studies

Variable	HHD (*n* = 23) Mean ± SD	Controls (*n* = 23) Mean ± SD	*p*- value
*Baseline characteristics*			
Birth year	1,964 ± 10 (1942–1984)	1,966 ± 11 (1947–1984)	
Female sex *n* (%)	16 (70%)	16 (70%)	
BMI, kg/m^2^	26.4 ± 3.8	26.0 ± 4.6	
Weight (kg)	77.2 ± 12.2	75.4 ± 17.7	
Height (cm)	170.7 ± 7.2	169.7 ± 13.4	
Current smoker (*n*)	1	2	
Diabetes family history (*n*)	6	7	
*Metabolic parameters*			
HbA1c (mmol/mol)	37.4 ± 6.0	35.8 ± 3.9	0.50
Proinsulin (pmol/L)	9.6 ± 10.0	6.6 ± 7.3	0.30
Insulin (mIE/L)	10.0 ± 6.8	9.2 ± 5.3	0.98
Proinsulin/insulin (ratio)	1.0 ± 0.6	0.7 ± 0.4	**0.05**
C-peptide (nmol/L)	0.8 ± 0.4	0.7 ± 0.2	0.90
Proinsulin/C-peptide (ratio)	11.1 ± 6.0	8.4 ± 5.1	0.11
HOMA2-%B	105.9 ± 36.7	109.6 ± 20.7	0.10
HOMA2-%S	66.2 ± 24.7	67.8 ± 24.9	0.91

BMI: body mass index (height/(weight*height)); HOMA: Homeostasis Model Assessment (B = β-cell function, S = insulin sensitivity); SD: standard deviation. Statistically significant difference is shown in bold.

### Statistical analyses

For the population-based study, odds ratios (OR) and 95% confidence intervals (95% CI) were estimated using a conditional logistic regression model. In this model, ORs can be regarded as risk ratios (RR), because of the incidence density matching procedure. Two-tailed Mann–Whitney *U* test was used for the clinical cohort analyses and *p*-value < 0.05 was considered significant. Analyses were conducted in GraphPad Prism (GraphPad Software Inc., San Diego, CA, USA) and SAS Version 9.4 (SAS Institute, Cary, NC, USA).

### Ethics approval

The study was approved by the Regional Ethics Committee in Stockholm (Dnr: 2019-01298).

## RESULTS

### Population-based cohort study

A total of 347 individuals with HHD were identified (55.9% females) and 34,700 comparison individuals (55.9% females). Due to the low incidence of diagnosis, the risk of T1D and other specified diabetes among individuals with HHD could not be modelled. Regarding T2D, individuals with HHD had a 1.2 times excess risk, but the confidence interval included the null (CI 0.9–1.8). The sex-stratified analysis, it was found that males with HHD had a 70% elevated risk of T2D (RR 1.7, CI 1.1–2.8), whereas no excess risk could be confirmed among women (RR 1.0, CI 0.5–2.0). All results are shown in **Table II**.

**Table II:** Risk ratios (RR) with corresponding 95% confidence intervals (95% CI) of the risk of type 1 diabetes, type 2 diabetes, and other specified diabetes in individuals with Hailey-Hailey disease (HHD) compared with individuals without HHD matched on birth year

Variable	Individuals with HHD *n* = 347 *n* (%)	Matched comparison subjects *n* = 34,700 *n* (%)	RR (CI)
Type 1 diabetes	6 (1.7)	736 (2.1)	n/a*
Males only	4 (2.6)	426 (2.8)	n/a*
Females only	2 (1.0)	310 (1.6)	n/a*
Type 2 diabetes	29 (8.4)	2,166 (6.2)	1.2 (0.9–1.8)
Males only	19 (12.4)	1,205 (7.9)	**1.7 (1.1–2.8)**
Females only	10 (5.2)	961 (4.9)	1.0 (0.5–2.0)
Other specified diabetes	7 (2.0)	481 (1.4)	n/a*

The risk of type 2 diabetes was also estimated separately for males and females.

Statistically significant RR are shown in bold.

* RR not calculated due to an insufficient number of individuals for the outcome.

n/a: not applicable.

### Family/pedigree studies

**Fig. 1:**
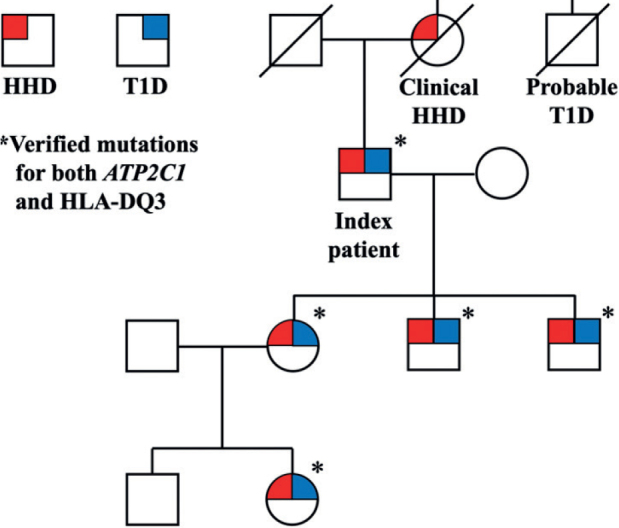
**Pedigree of the family with *ATP2C1*-mutation verified Hailey-Hailey disease (HHD), type 1 diabetes (T1D) and HLA-DQ3 mutations.** *Circles*: females; *squares*: males.

*Family with type 1 diabetes and Hailey-Hailey disease.* Five patients with biopsy-proven HHD were evaluated at the Department of Dermato-Venereology at Karolinska University Hospital, Stockholm, Sweden (*Fig. 1). The index patient was a male born in 1951 with a diagnosis of HHD since 20 years of age and T1D since 23 years of age. The index patient’s 3 children all inherited their father’s HHD and were diagnosed between the ages of 20 and 30. All individuals shared the same missense mutation in *ATP2C1*, namely c.1730C > T (p.Ala577Glu). To our knowledge, this mutation is previously unpublished (19). They were also all diagnosed with fully insulin-dependent T1D in the age range 13–23 years, positive for anti-zinc transporter 8 antibodies and anti-tyrosine phosphatase-like insulinoma antigen 2 (IA-2) antibodies, but negative for anti-glutamic acid decarboxylase (GAD) antibodies. Further analysis revealed that all 4 patients had a mutation in HLA-DQ3, a high-risk allele for the development of T1D, ranging from 5% in siblings to 55% in identical twins (20, 21). The granddaughter of the index patient also shared the same mutation in *ATP2C1* and HLA-DQ3 as well as a diagnosis of T1D, albeit without having debuted with any skin symptoms, possibly due to young age (16 years of age). The pedigree shows a complete inheritance rate for T1D, which is far higher than the risks reported in the literature (4.9 ± 1.7%) (22).

**Fig. 2:**
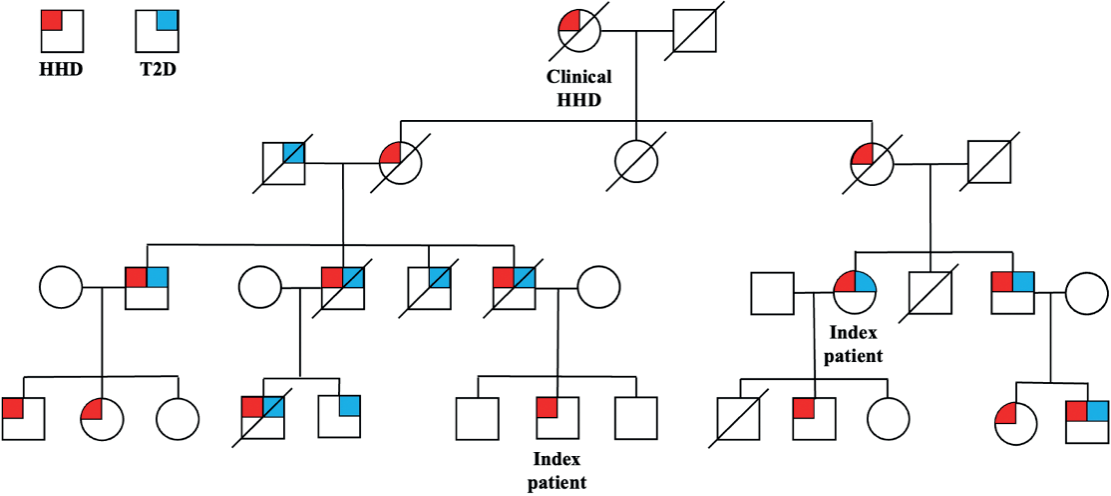
**Pedigree of the family with Hailey-Hailey disease (HHD) and type 2 diabetes (T2D).** *Circles*: females; *squares*: males.

*Family with type 2 diabetes and Hailey-Hailey disease.* The study reviewed 4 generations of a family with a high co-aggregation of HHD and T2D (*Fig. 2). Family history was obtained from 2 index patients and medical records. Diagnosis of HHD was made on the basis of typical clinical appearance, family history, and histopathology. Overall, a higher degree of inheritance for T2D was seen than the expected lifetime risk of 40% if 1 parent has T2D or 70% if both parents have T2D (22). Limitations are the lack of data on where T2D might have entered on the right side of the pedigree, and that all individuals in the youngest generation are still below the usual age of T2D onset (23).

### Clinical cohort study

Individuals with HHD displayed a significant increase in proinsulin/insulin ratio compared with healthy controls (Table I). Differences in HbA1c, proinsulin, insulin, C-peptide, proinsulin/C-peptide ratio, HOMA2-%B (pancreatic β-cell function), and HOMA2-%S (insulin sensitivity) were statistically insignificant.

## DISCUSSION

By using a population-based cohort design, a matched clinical patient-control cohort, and analyses of 2 separate family pedigrees, this study presents the first evidence of an association between HHD and diabetes. This discovery represents the first described extracutaneous comorbidity of HHD and underscores the importance of cellular Ca^2+^ homeostasis in diabetes pathophysiology.

This study revealed that individuals with HHD exhibit a 1.2 times heightened risk for T2D, although the increased risk is not statistically significant. It is notable that male patients with HHD displayed a significant 70% elevated risk. In addition, study of a family with HHD mutations in both *ATP2C1* and HLA-DQ3 suggests a potential synergistic risk increase for T1D. A separate HHD family showcased an unusual inheritance pattern for T2D, supporting the hypothesis of HHD being a synergistic risk factor for diabetes.

### Calcium homeostasis in Hailey-Hailey disease and diabetes

Ca^2+^ has a crucial role in the skin, namely in keratinocyte differentiation, adhesion, motility and lipid secretion (24). Ca^2+^ concentration in the Golgi lumen is significantly lower in patients with HHD than in healthy individuals (2). Such dysregulation of Ca^2+^ homeostasis in HHD can have many implications for diabetes.

Ca^2+^ dysregulation may contribute to T1D, in that changes in intracellular Ca^2+^ levels alter post-translational modification of proteins (25), which enhances their immunogenic properties (26) leading to the generation of neo-autoantigens, which could stimulate autoimmune β-cell destruction, a direct cause of T1D (27). This process has also been shown to occur for proinsulin per se (28). Regarding HLA-DQ3, it is considered a high-risk gene for developing T1D, as approximately 5% of individuals with this HLA genotype are diagnosed with T1D by age 15 years, in comparison with only 0.3% of the general population (9). Still, most patients in the general population with HLA-DQ3 mutations do not develop T1D. Therefore, it is extraordinary that all family members in the current study pedigree (Fig. 1) developed T1D, suggesting that HHD might act as a synergistic risk factor.

It is well established that low levels of intracellular Ca^2+^ in several tissues, such as skeletal muscle and adipose tissue are associated with T2D pathophysiology (29), and normal Ca^2+^ signalling is essential for insulin secretion (30). An increased proinsulin/insulin ratio, as seen in the current clinical HHD cohort, has been previously associated with a T2D metabolic phenotype (31, 32). Thus, the current results are in congruence with evidence indicating an underlying biological rationale for the association between HHD and T2D.

Darier disease, akin to HHD due to a similar cellular phenotype stemming from mutations in *ATP2A2*, was found to have an increased risk of T1D (33). The shared cellular phenotype might explain the observed metabolic indications for T2D in patients with Darier disease (18).

### Strengths and limitations

This study has several strengths. The data in the cohort study comes from a linkage of unique, longitudinal Swedish nationwide registers, well-known for their quality and usefulness for medical research. Moreover, all diagnoses were assigned by medical doctors in specialist care settings, and the sample size is particularly large. The study also explored possible associations from the standpoints of a clinical cohort and individual family pedigrees. The population-based cohort study has limitations that should be considered when interpreting the results: there was a lack of sufficient statistical power for T1D and other specified diabetes in both sexes, and for females, there was a lack of sufficient power for T2D. The clinical cohort was relative-ly small due to the rarity of HHD, possibly leading to issues regarding statistical power. Larger register-based or clinical cohort studies are warranted to substantiate these findings.

### Conclusion

This study presents, for the first time, an association between HHD and diabetes. In particular, male individuals with HHD had a 70% elevated risk of T2D. This study presents 2 family pedigrees that highlight the potential effect of HHD as a synergistic risk factor in the development of diabetes. Patients with HHD also display higher proinsulin/insulin ratios indicative of a T2D metabolic phenotype. Further investigation into the association between T1D and HHD is warranted, as our population-based sample contained an insufficient number of patients for analysis.

These findings highlight the potential importance of SPCA1, the Golgi apparatus, and cellular Ca^2+^ homeostasis in diabetes pathophysiology. We propose that HHD may enhance the risk of diabetes development. Further research into patients with HHD could aid in early diagnosis and treatment of T1D and T2D, and genetic counselling and possible prenatal diagnosis regarding both HHD and diabetes risk would be of great benefit to patients.
